# Artesunate inhibits airway remodeling in asthma *via* the MAPK signaling pathway

**DOI:** 10.3389/fphar.2023.1145188

**Published:** 2023-03-14

**Authors:** Mengyuan Zhang, Jiangtao Lin, Jingyuan Zhang, Ruiheng Zhao, Jingxuan Wan, Ying Nong

**Affiliations:** ^1^ Department of Respiratory and Critical Care, China-Japan Friendship Hospital, Beijing, China; ^2^ Graduate School of Chinese Academy of Medical Sciences, Peking Union Medicine College, Beijing, China; ^3^ Department of Respiratory and Critical Care, Beijing Shijitan Hospital, Capital Medical University, Beijing, China; ^4^ Graduate School of Beijing University of Chinese Medicine, Beijing, China; ^5^ Department of Respiratory and Critical Care Medicine, The First Affiliated Hospital of Nanchang University, Beijing, China

**Keywords:** asthma, artesunate, FIZZ1, MAPK pathways, airway remodeling

## Abstract

**Background:** Artesunate (ART), is a semi-synthetic water-soluble artemisinin derivative extracted from the plant *Artemisia annua*, which is often used to treating malaria. *In vivo* and *in vitro* studies suggested it may help decrease inflammation and attenuate airway remodeling in asthma. However, its underlying mechanism of action is not elucidated yet. Herein, an attempt is made to investigate the ART molecular mechanism in treating asthma.

**Methods:** The BALB/c female mice sensitized *via* ovalbumin (OVA) have been utilized to establish the asthma model, followed by carrying out ART interventions. Lung inflammation scores by Haematoxylin and Eosin (H&E), goblet cell hyperplasia grade by Periodic Acid-Schiff (PAS), and collagen fibers deposition by Masson trichrome staining have been utilized for evaluating how ART affected asthma. RNA-sequencing (RNA-seq) analyses were performed to identify differentially expressed genes (DEGs). The DEGs were analyzed by Gene Ontology (GO) terms, Kyoto Encyclopedia of Genes and Genomes (KEGG) pathways, and Protein-Protein interaction (PPI) function analyses. Hub clusters were found by Cytoscape MCODE. Subsequently, Real-Time quantitative PCR (RT-qPCR) verified the mRNA expression profiles of DEGs. Finally, immunohistochemistry (IHC) and western blots have validated the relevant genes and potential pathways.

**Results:** ART considerably attenuated inflammatory cell infiltration, mucus secretion, and collagen fibers deposition. KEGG pathway analysis revealed that the ART played a protective role *via* various pathways including the mitogen-activated protein kinase (MAPK) pathway as one of them. Moreover, ART could alleviate the overexpression of found in inflammatory zone 1(FIZZ1) as revealed by IHC and Western blot analyses. ART attenuated OVA-induced asthma by downregulating phosphorylated p38 MAPK.

**Conclusion:** ART exerted a protective function in a multitarget and multi-pathway on asthma. FIZZ1 was a possible target for asthma airway remodeling. The MARK pathway was one of the key pathways by which ART protected against asthma.

## 1 Introduction

Asthma is a common chronic inflammatory disease of the airways that affects at least a 300 million people worldwide (Soriano et al., 2017). It is defined by heterogeneous respiratory syndrome such as cough, wheezing, shortness of breath, and chest tightness. Among chronic respiratory disorders, asthma remained the second most common and the second leading contributor to death globally (Collaborators, 2020). Even though many developed nations have seen a constant or declining asthma rate, it is sweeping across developing nations (Papi et al., 2018).

Asthma is usually characterized by airway hyperresponsiveness (AHR), airway inflammation, and airway remodeling. Exogenous allergens (pollens, viruses, fungi, or dust mites) can cause T cells to become activated (Holgate, 2012) by antigen-presenting from dendritic cells or macrophages. Interleukins such as IL-4, IL-5, and IL-13 are produced by activated T helper 2 (Th2) cells (Bowen et al., 2008), which induces B cells to generate immunoglobulin E (IgE), which can bind to the surface of mast cells (Li-Weber and Krammer, 2003). The interaction of allergen and IgE causes mast cells to produce active mediators, which lead to airway smooth muscle contraction (Palmer et al., 2001), increased mucus secretion, and inflammatory cell infiltration (Bingham and Austen, 2000). Additionally, Th2 cytokines can directly activate mast cells, eosinophils, and macrophages, causing them to assemble around airways (Hammad and Lambrecht, 2021). Additional inflammatory chemokines secreted by the aforementioned cells, establish a complex airway inflammation network with inflammatory cells together, which causes airway hyperresponsiveness (Cockcroft and Davis, 2006). Besides, it is thought that Th1/Th17 cells contribute significantly to steroid-resistant asthma and severe asthma, which are primarily characterized by neutrophil infiltration (Wenzel, 2012). Chronic airway inflammation results in a constant injury-repair process of the airway, which can cause airway remodeling manifestations. Airway remodeling mostly refers to the alterations of airways wall structure (Vignola et al., 2003). Airway wall thickening is the main manifestation (Bergeron and Boulet, 2006). It mainly consists of airway epithelial cell mucus metaplasia (Holgate, 2007), epithelial-mesenchymal transition (EMT) (Eiwegger and Akdis, 2011), deposition of extracellular matrix protein (ECMs) stimulated by epithelial cells, thickening of basement membrane (Guida and Riccio, 2019), smooth muscle hypertrophy/hyperplasia (Joubert and Hamid, 2005), and vascular hyperplasia (McDonald, 2001), etc. Airway remodeling can present early in childhood, suggesting it is not simply a consequence of inflammation (O'Reilly et al., 2013). Asthma patients who undergo airway remodeling will have permanent airflow restrictions airflow obstruction (Lemanske and Busse, 2003). One of the most important goals of asthma therapy is avoiding complications such as airway remodeling. Glucocorticoids, beta-2 receptor agonists, anticholinergic drugs, and biological therapies (Hurtado et al., 2020) were the mainstays of asthma care when they were both available and affordable (https://ginasthma.org/reports/). Although regular diagnosis and treatment can alleviate symptoms for the vast majority of asthma patients, those with severe asthma may require long-term inhalation of medium-high doses of glucocorticoids or even oral large doses of glucocorticoids, both of which carry the risk of developing undesirable side effects. Some patients do not respond well to hormone therapy and have to choose biological inhibitors, which may cause serious social and economic burdens. Meanwhile, the above drugs cannot completely control airway remodeling, making the airflow restriction irreversible. Therefore, there is necessary for searching a safe and economic drug to treat asthma and even relieve airway remodeling.

**FIGURE 1:**
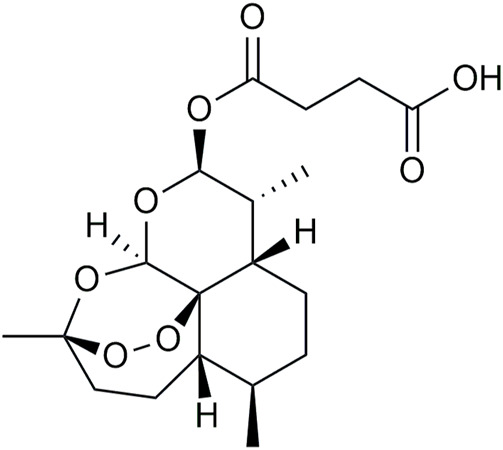
Chemical structure of Artesunate.

Artesunate (ART) **(** Figure 1 **)** is a semi-synthetic water-soluble artemisinin derivative with high activation and rare toxicity. It has been extracted utilizing *Artemisia annua* by Chinese scientist Tu Youyou, which possessed poor bioavailability and limited effectiveness (Sun et al., 2019). To meet the clinical requirements, some derivatives of artemisinin have been developed including ART (Zhang et al., 2022). ART was initially used for antimalarial treatment (Arguin et al., 2008). After extensive study, ART was found to attenuate inflammation (Feng and Qiu, 2018; Mancuso et al., 2021), inhibit cancer (Sun et al., 2019; Zhao et al., 2020; Li et al., 2021), alleviate fibrosis (Kong et al., 2019), promote ischemia and reperfusion injury (Zhang et al., 2020), inhibit rabies virus (Luo et al., 2021), and SARS-CoV-2 (Hu et al., 2021) replication. Especially in asthma, ART could alleviate airway wall remodeling by inhibiting airway smooth muscle cell proliferation (Tan et al., 2014) as a combination therapy with glucocorticoids. By preventing IgE-induced mast cell degranulation, it may potentially exert anti-allergic effects (Cheng et al., 2013). Meanwhile, it was also reported to ameliorate oxidative damage in allergic airways and suppressed OVA-induced eosinophil counts increases in bronchoalveolar lavage fluid (BALF) cells (Ho et al., 2012). Our earlier research found that ART could reverse glucocorticoid insensitivity by inhibiting the Phosphoinositide 3-Kinase (PI3K)/Protein Kinase B(PKB, also AKT) pathway (Luo et al., 2015), reduce eosinophil infiltration, and promote eosinophil apoptosis in asthmatic mice (Wang et al., 2020).

At present, multiple studies have displayed ART’s potential value in treating asthma, but their research interests often focus on one specific cell or one target gene (Ho et al., 2012; Cheng et al., 2013; Tan et al., 2014; Luo et al., 2015; Wang R. Y. et al., 2019; Wang Y. et al., 2019). There is no systematic description of the mechanism, so it is worthwhile to explore the corresponding mechanisms (Zhang et al., 2022).

Using an OVA-induced asthma mouse model, transcriptomic RNA sequencing (RNA-seq) (Tanner et al., 2022) was conducted to explore ART’s biological mechanism of action.

## 2 Materials and methods

### 2.1 Animals and treatments

**FIGURE 2:**
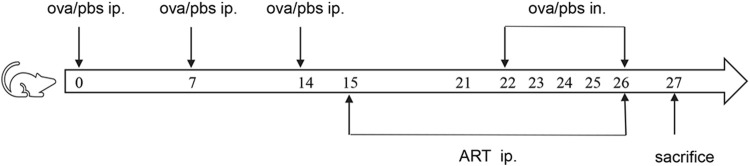
The establishment of mouse asthma model.

Female BALB/c mice aged 6–7 weeks without specific pathogens (SPF), weighing 16–18 g, have been procured from Vital River Laboratory Animal Technology Co., Ltd. (Beijing, China) and placed in a pathogen-free environment at the clinical research institute of China-Japan Friendship Hospital (Beijing, China). The experimental animals have been randomly classified into four groups viz (Table 1): the control group (CONTROL), the ovalbumin group (OVA), the Artesunate + OVA group (ART + OVA), the Artesunate + control group (ART + CONTROL). Both the OVA and ART + OVA groups have been subjected to sensitization utilizing intraperitoneal administration of OVA suspension (100 μg) (Sigma-Aldrich, St. Louis, MO, United States; grade V) and aluminum hydroxide (2.25 mg) in phosphate buffer saline (PBS) (200 µL per mouse) on days 0, 7, and 14, whereas the CONTROL, as well as the ART + CONTROL groups, were given an intraperitoneal injection of equivalent volumes of PBS. Both the ART + OVA and the ART + CONTROL groups were given Artesunate (30 mg/kg, Sigma-Aldrich, St. Louis, MO, United States, dissolved in 7.5% NaHCO3, diluted with PBS) by intraperitoneal injection during 15–21 days. Both the CONTROL as well as the OVA groups have been injected with equal PBS volume at the same time. Then the mice were challenged by inhaled OVA (100 µg in 50 ul PBS) (as the OVA and the ART + OVA groups) or equivalent PBS (as the CONTROL and the ART + CONTROL groups) from Days 22 to 26. ART (groups: ART + OVA and ART + CONTROL) or PBS (groups: CONTROL and OVA) was intraperitoneally injected 1 h before the challenge. The body weight of mice in each group was measured 24 h after the last challenge, and each mouse was euthanized (Figure 2).

**TABLE 1:** Method of establishing mice model of asthma.

Groups	Sensitization	Drug intervention	Challenge
	Day 0, 7, 14	Day 15–26	Day 22–26
CONTROL	PBS	PBS	PBS
OVA	OVA	PBS	OVA
ART + OVA	OVA	ART	OVA
ART + CONTROL	PBS	ART	PBS

### 2.2 Assessment of airway hyper-responsiveness

Mice in each group were weighed 24 h after the final stimulation, and then they were anesthetized by intraperitoneal injection of pentobarbital sodium (50 mg/kg). Tracheotomy and endotracheal intubation were then conducted. Airway hyper-responsiveness to methacholine (Mch, Sigma-Aldrich) was assessed by the FlexiVent system (SCIREQ, Inc., Montreal, Canada) (Wang et al., 2021). Methacholine solution was dripped into the atomizing pump from low to high concentration (0, 3.125, 6.25, 12.5, 25, 50 mg/mL) consecutively. The respiratory resistance (Rrs) was measured after 30 s of atomization, within 5 min of atomization, and the average detected value was taken to as the Rrs value under this excitation concentration. Then the values were used to analyze the AHR.

### 2.3 Lung histopathology

Left lung lobes were immersed in 4% paraformaldehyde for 24 h and then embedded in paraffin. The paraffin-embedded sections (4 µm) have been stained utilizing Haematoxylin and Eosin (H&E), Periodic Acid-Schiff (PAS), and Masson trichrome staining.

H&E was employed for assessing the inflammation of the lungs. The degree of inflammatory cell infiltration surrounding the tiny airways was used to quantify the level of inflammation, which was rated on a scale from 0 to 4. The criteria (Brassard et al., 2021) were as follows: 0, no cells; 1, a little inflammatory cell infiltration around the airway; 2, 1-2 layers of inflammatory cells infiltrating around the airway; three points, 3-5 layers of infiltration; 4, more than five layers of infiltration. Inflammation scores were completed by one investigator in a single-blind manner. There are eight bronchioles counted in per slide utilizing Case Viewer software (version 1.3; 3D Histech, Budapest, Hungary).

The hyperplasia of goblet cells has been examined utilizing PAS staining with the following grading system (Padrid et al., 1995): 0, goblet cells absent; 1, goblet cells <25% of epithelial lining cells; 2, goblet cells in 25%–50%; 3, goblet cells in 50%–75%; 4, goblet cells >75%. The PAS scores were computed by one study-blinded investigator. Eight bronchioles were counted in each specimen utilizing a light Axiovert ×200 microscope (Carl Zeiss GmbH, Jena, Germany).

Masson staining has been applied for assessing the collagen fibers deposited in airways, which will be stained blue. Then the percentage of collagen fibers areas were calculated by Image J software (NIH, Bethesda, MD) (Dong et al., 2021). Each mouse had at least eight bronchioles counted by measuring the blue-stained region.

### 2.4 Transcriptomics analysis

Transcriptomics RNA sequencing has been carried out by Wekemo Tech Group Co., Ltd. (Shenzhen, China). Considering that the drug will not be used on healthy people in practical application, total RNAs of 50 mg lung regions were isolated from the three groups: CONTROL (*n* = 3, randomly), OVA (*n* = 3, randomly), and ART + OVA (*n* = 4, randomly) group for the transcriptional analysis. Genes possessing | log2 (fold change, Fc) | > 1 as well as a significant *p*-value less than 0.05 as evaluated by DESeq2 (1.16.1) have been assigned as DEGs between two groups. The intersection of DEGs between the two groups was taken. The (GO) and (KEGG) pathway enrichment of DEGs have been performed through the Functional Annotation Bioinformatics Microarray Analysis (DAVID) database (Li et al., 2019; Sherman et al., 2022) (https://david.ncifcrf.gov/). GO includes biological processes (BP), cellular components (CC), and molecular function (MF). GO and KEGG terms with *p* < 0.05 were considered significantly enriched by DEGs. STRING (v 11.5) (https://cn.string-db.org/) has been applied for constructing PPI function analyses for the proteins encoded by intersecting DEGs, eventually resulting a PPI network. Cytoscape (version 3.9.1) was used to integrate data, analyze, and visualize the above PPI network, and then we screened the core modules out using the Molecular Complex Detection (MCODE) plug-in.

### 2.5 Real-Time quantitative PCR (RT-qPCR)

The total RNA has been extracted from lung tissue samples using RNAiso Plus (9109, Takara) following the manufacturer’s instruction and then reverse-transcribed into cDNA utilizing PrimeScript™ RT Master Mix (RR036A, Takara). Then Real-time PCR was performed with TB Green Premix Ex Taq II (RR820A, Takara) using Applied Biosystems 7500 Real-Time PCR System (Thermo Fisher Scientific), which exported Ct values. The Relative mRNA expression level was evaluated by 2^−ΔΔCT^ method (Livak and Schmittgen, 2001), which based on the assumption that the target gene had the equal amplification efficiency as the reference gene (GAPDH). By comparing the relative quantification of target gene and reference gene between different groups, the differences were determined.

$$∆∆Ct={\left({Ct}_{target}-{Ct}_{GAPDH}\right)}_{sample}-{\left({Ct}_{target}-{Ct}_{GAPDH}\right)}_{control}$$

PCR primer sequences were listed in Table 2.

**TABLE 2:** Nucleotide sequences of primers used in RT-qPCR.

Gene	Primer	Sequence (5′-3′)
IL5RA	Forword	GGT​TGT​CTC​CTG​CGA​CTT​CA
	Reverse	TGG​TCC​AGG​GTT​TCT​TAC​TCC
CCR3	Forword	AAG​GTG​GAG​AGT​GAC​TAG​GCA​GAT​C
	Reverse	GGG​AAT​GGT​GTC​TTT​GTG​TTG​TGT​G
IL13RA2	Forword	GGA​GCG​AAT​GGA​GTG​AAG​AGG​AAT​G
	Reverse	CTG​CTG​GCT​GGC​TCT​ATG​TCA​AG
TNFRSF8	Forword	CCA​GCA​CGG​GAC​ACA​AGT​TGA​G
	Reverse	CAT​CCA​GCA​GCG​GCA​GGT​TC

### 2.6 Immunohistochemistry

Immunohistochemistry has been applied for determining where the FIZZ1 protein was localized and its concentration. Lung paraffin sections were deparaffinized, rehydrated, and blocked by 3% H_2_O_2_. Sections were then blocked with goat serum at a concentration of 5% to reduce the absorption of non-specific immunoglobulins. After that, the specimens were incubated with anti-FIZZ1 antibody (1:1,000, ab39626, Abcam) at 37°C for 2 h, followed by incubating with a secondary antibody at 37°C for 30 min. Image-Pro Plus software (Version X; Adobe, San Jose, CA) has been applied for evaluating protein expression. In total, eight images of bronchioles from each tissue section were examined using ×400 magnification and the program Case Viewer (version 1.3; 3DHistech, Budapest, Hungary). Data were presented as an average optical density (AOD) for the entire sections of lung tissue showing positive staining.

### 2.7 Western blot

The proteins from lung tissues have been extracted utilizing the RIPA Lysis Buffer containing protease inhibitor (GRF101, Epizyme) and phosphatase inhibitor cocktails (GRF102, Epizyme) while quantification performed utilizing a BCA Protein Assay Kit (ZJ101, Epizyme). Equivalent amounts of proteins have been electrophoresed on polyacrylamide gels and electrotransferred onto polyvinylidene fluoride (PVDF) membranes. The membranes were incubated for 2 h at room temperature by the primary antibodies which included *ß*-Tubulin antibody (HX 1829, huaxingbio), anti-FIZZ1 antibody (1:1,000, ab39626, Abcam) p38 mitogen-activated protein kinase (MAPK) Rabbit mAb (#8690, Cell Signaling Technology), Phospho-p38 MAPK (Thr180/Tyr182) Rabbit mAb (#4511, Cell Signaling Technology). Following a 40-min incubation at room temperature with secondary antibodies, the membranes were detected using a chemiluminescence substrate system (Bio-Rad Laboratories, CA, United States). The band intensities of proteins have been quantitated using Image J software.

### 2.8 Statistical analysis

All data were analyzed by GraphPad Prism 9.3 software (San Diego, CA, United States) using non-parametric tests. Measurement data were expressed as mean ± standard deviation (mean ± SD). When comparing multiple groups, ordinary one-way analysis of variance (ANOVA) followed by Tukey’s test, was used to perform significant differences. The variations were assumed statistically significant when *p* < 0.05.

## 3 Results

### 3.1 The behavioral change of mice

The asthma model OVA group and drug ART + OVA group mice were restless and irritable, accompanied by breathing hard, erected neck hair, and scratchy and uneasy forelimbs after OVA stimulation. The above symptoms and signs can partially relieve after 30 min when the mice were quiet in the cage. The CONTROL and ART + CONTROL groups mice did not show the above signs. Based on the observed alteration in participant behavior, it is tentatively concluded that the asthma model had been effectively developed.

### 3.2 ART attenuated OVA-induced AHR

**FIGURE 3:**
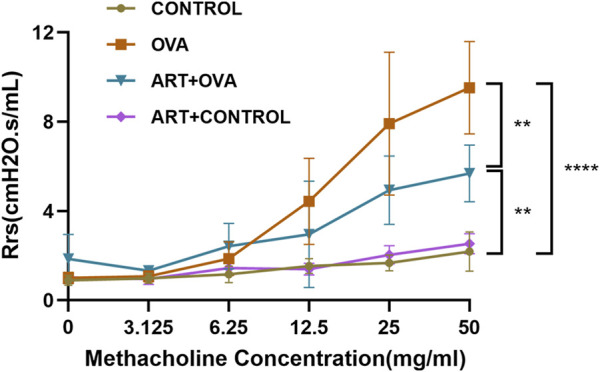
The effect of ART on airway hyperresponsiveness in OVA-induced asthma mice. **p < 0.01, ****p < 0.0001.

To determine the effect of ART on AHR, we conducted the bronchial provocation test to measure the Rrs. As shown in Figure 3, when the concentration of acetylcholine increased, the Rrs had no significant change in CONTROL and ART + CONTROL groups, while increased in OVA and ART + OVA groups. When the concentration reached 50 mg/mL, the airway resistance of OVA group was significantly higher than CONTROL group (*p* < 0.0001). After treatment with ART, the airway resistance of mice decreased considerably (*p* < 0.01), compared with OVA group.

### 3.3 ART reduced OVA-induced lung inflammation, goblet cell hyperplasia, and airway remodeling

**FIGURE 4:**
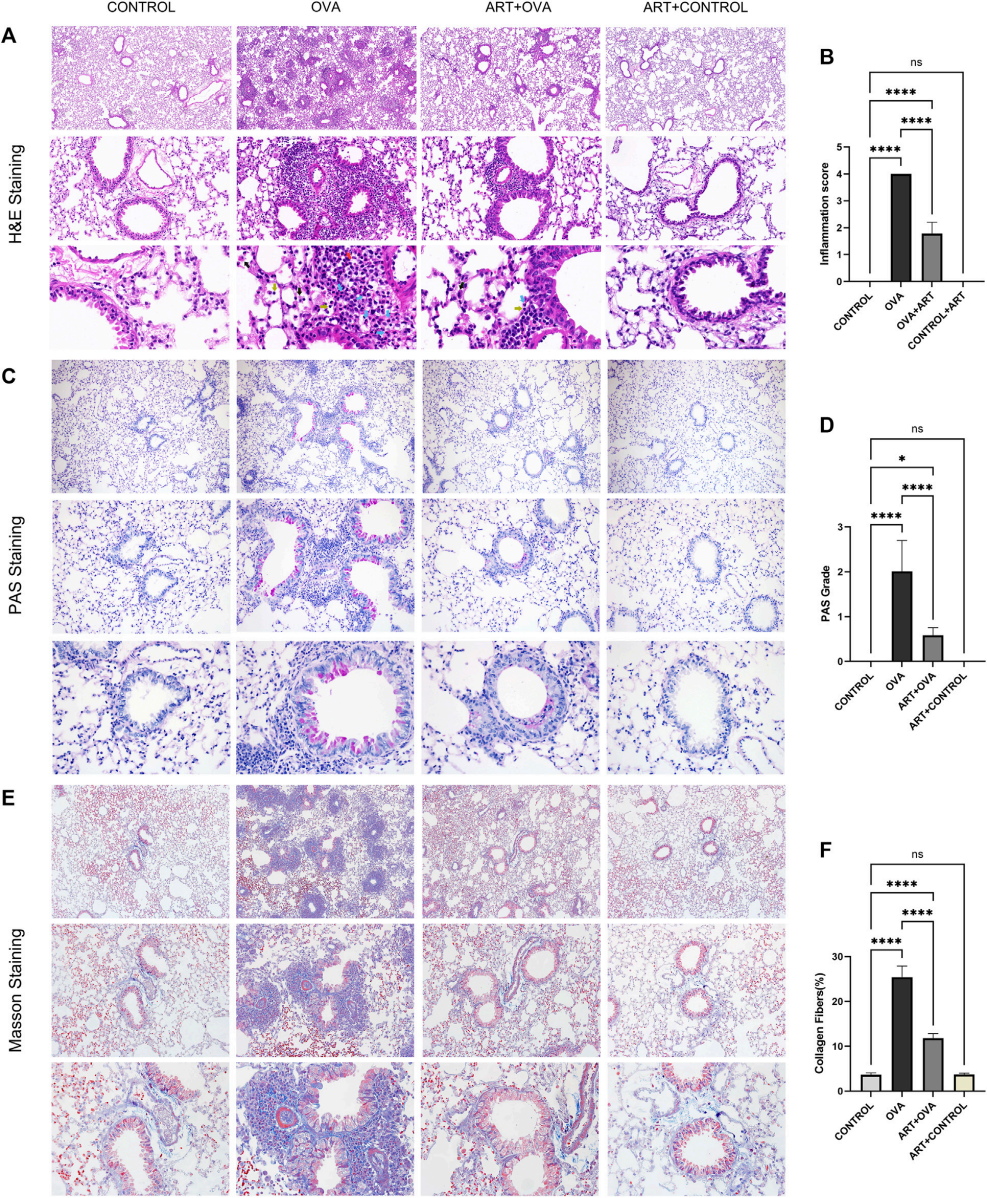
Effects of ART treating OVA-induced asthma in mice. **(A)**, Representative Images of HE (Images were captured at ×100, ×400, and ×1000 magnification). Blue arrows, yellow arrows, black arrows and red arrows refer to eosinophils, lymphocytes, macrophages, and neutrophils respectively. **(C,E)**, PAS and Masson staining from lung sections each group (Images were captured at ×100, ×200, and ×400 magnification). **(B,D,F)** The inflammatory infiltration, goblet cell hyperplasia and Masson positive area were quantified by inflammation scores, PAS scores and percentage of collagen fibers (*n* = 6). *p < 0.05, **p < 0.01, ***p < 0.001, ****p < 0.0001.

In comparison to the CONTROL group, the OVA group’s inflammatory cells significantly infiltrated the peribronchiolar, perivascular, alveolar interval, and alveolar cavity, where eosinophils were predominant, macrophages, lymphocytes, and a little quantity of neutrophils could also be observed. Thickening of the bronchial wall, and swelling of airway epithelium, could also be found in the OVA group. The aforementioned infiltration was suppressed by ART, with a lower HE scores compared to the OVA-treated group (Brassard et al., 2021) (Figures 4A, B). Meanwhile, mice with OVA showed a significantly obvious mucus production in epithelial layers. PAS staining specimens of the ART + OVA group revealed that the score of PAS was lower compared with the OVA group (Figures 4C, D). Results gained from Masson’s staining suggested collagen fiber accumulation increased around airways in the OVA group, compared with the CONTROL group. Moreover, compared with the OVA group, the collagen around the airway was less in the ART + OVA group (Figures 4E, F). Masson staining demonstrated that ART administration prevented lung remodeling in OVA-induced mice. Overall, the pathological changes of ART-treated mice showed more amelioration than asthma model group, but not completely attenuated when compared with the control mice.

### 3.4 RNA-seq transcriptomics in ART-treated OVA-induced asthma

**FIGURE 5:**
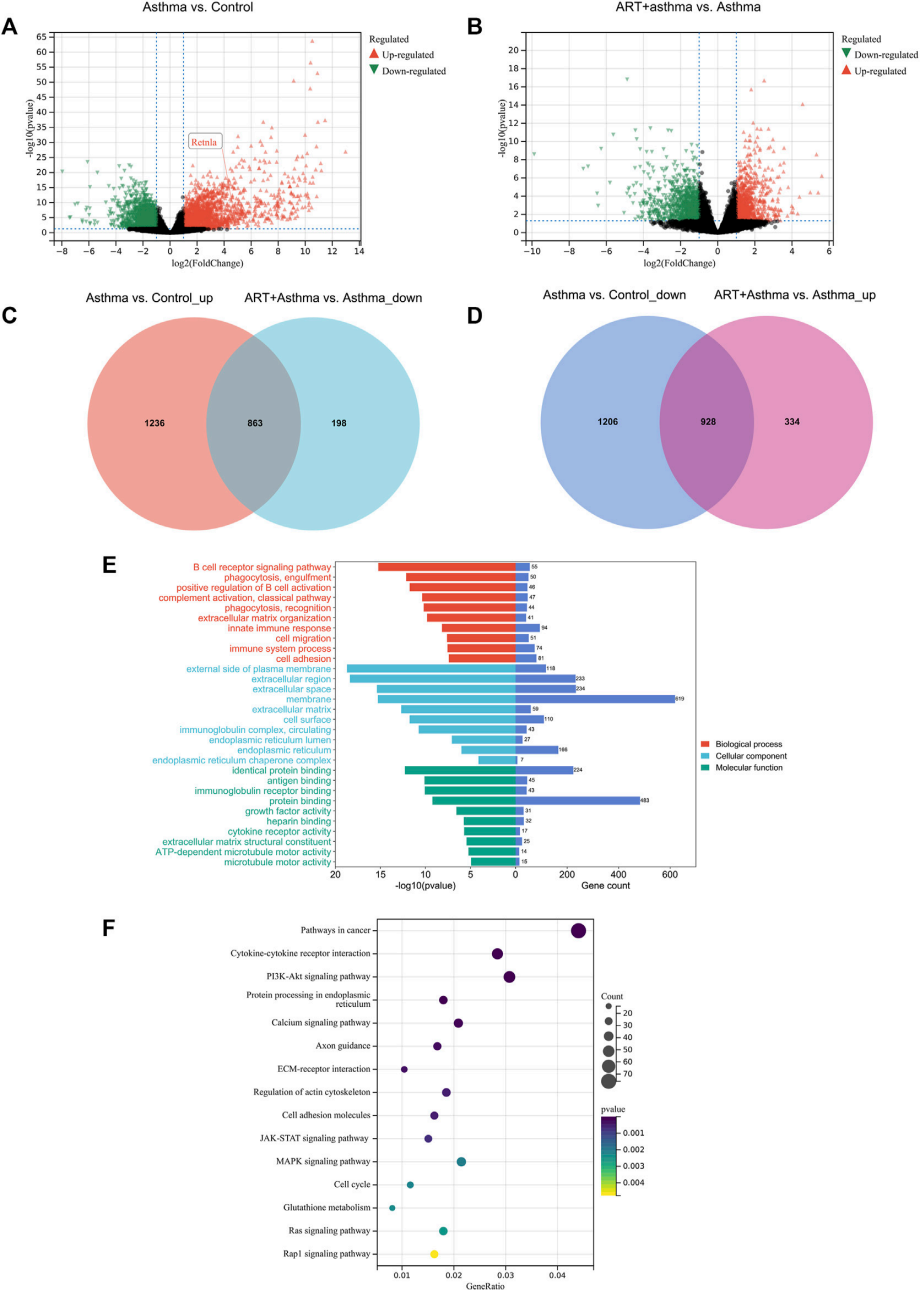
Transcriptional analysis of ART against Asthma by RNA-seq technology. **(A,B)** Volcano plot of differentially expressed genes (DEGs) between Asthma vs. Control, and ART + asthma vs. Asthma based on RNA-seq results. **(C,D)** Venn diagram of upregulated and downregulated DEGs. **(E,F)** GO and pathway enrichment analysis of the DEGs between ART + asthma and Asthma groups.

Analyzing the effects of ART on OVA-induced asthma at the molecular level required the use of transcriptomics to identify DEGs across the control, asthma, and ART groups. As per the screening criteria of |log2 (Fc) | > 1 as well as a significant *p*-value < 0.05 (as compared with the control group), 4,233 genes in the asthma group showed significant changes, out of which 2099 were upregulated, while 2,134 were downregulated. ART intervention substantially altered 2,323 genes compared to the asthma group, with 1,262 genes upregulated and 1,061 genes downregulated. Volcano plots showing the upregulated and downregulated genes among Asthma vs. Control and ART + asthma vs. Asthma groups have been illustrated (Figures 5A, B). ART could reverse 863 upregulated as well as 928 downregulated genes during OVA induction as illustrated by the Venn diagram of DEGs (Figures 5C, D). To further investigate the mechanisms of ART, GO enrichment as well as KEGG signaling pathway analyses have been conducted on DEGs of ART + Asthma vs. Asthma group. Compared with the asthma group, the terms of GO in the ART + Asthma group were mainly the B cell receptor signaling pathway, extracellular matrix organization, and immune system process. More detailed information on these is illustrated in Figure 5E. According to the *p*-value < 0.05, the top 15 pathways were demonstrated as the main pathways as shown in Figure 5F. The results show that the KEGG pathways of ART on asthma mainly included pathways in cancer, cytokine-cytokine receptor interaction, PI3K/AKT signaling pathway, JAK-STAT signaling pathway, MAPK signaling pathway, and so on. The most enriched gene pathway is pathways in cancer, which had 76 genes. Besides the pathways in cancer, the cytokine-cytokine receptor pathway was most closely associated with ART treatment of asthma.

**FIGURE 6:**
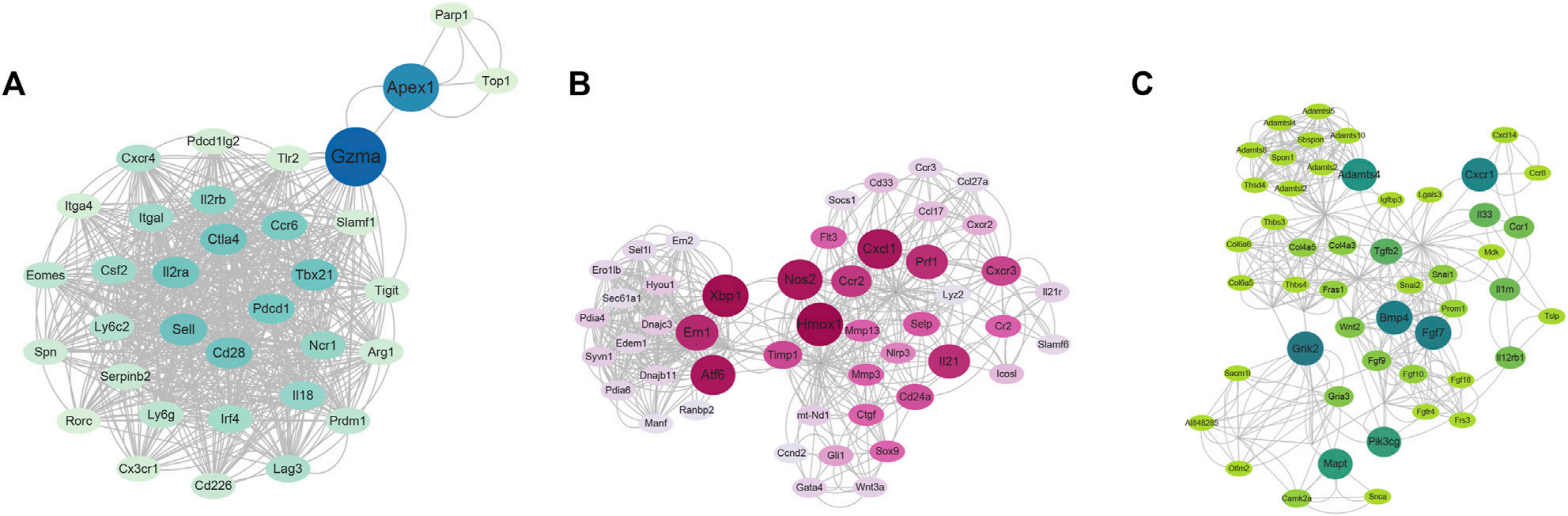
The clusters using MCODE *via* Cytoscape software based on RNA-seq results. **(A)** The cluster 2. **(B)** The cluster 3. **(C)** The cluster 4. (The nodes color and size were arranged by betweenness value, cutoff = 2, node score cutoff = 0.2, k-core = 2, and max. Depth = 100).

Also, a PPI network of DEGs between ART + Asthma and Asthma groups has been constructed by the STRING database. The network performed secondary processing by using Cytoscape. Large PPI networks have densely linked areas that may represent molecular complexes, which were identified using MCODE (Bader and Hogue, 2003). It has been shown that highly-connected regions of the PPI network (molecular complexes) have a higher probability of being involved in biological regulation. A total of 41 clusters were screened. Taking score >7 and node >30 as a standard, there were four clusters with MCODE. Cluster 1 mainly was related to mitosis. Cluster 2 mainly included leukocyte activation and migration, adaptive immune response, activation and differentiation of T cells, regulation of cytokine production, and cytokine-mediated signaling pathway (Figure 6A). Cluster 3’s primary biological functions were the control of inflammation, cytokine synthesis, leukocyte-mediated immunity, and B cell development (Figure 6B). Extracellular matrix structure, angiogenesis regulation, inflammatory response positive regulation, tissue remodeling regulation, smooth muscle cell migration control, and so on were all covered under Cluster 4 (Figure 6C). The other three clusters, except the first, may be explained by the immune system, inflammation, or the remodeling process. As a result of the aforementioned biological processes, ART was more likely to have a role in treating asthma.

### 3.5 Quantitative Real-time PCR (qPCR)

**FIGURE 7:**
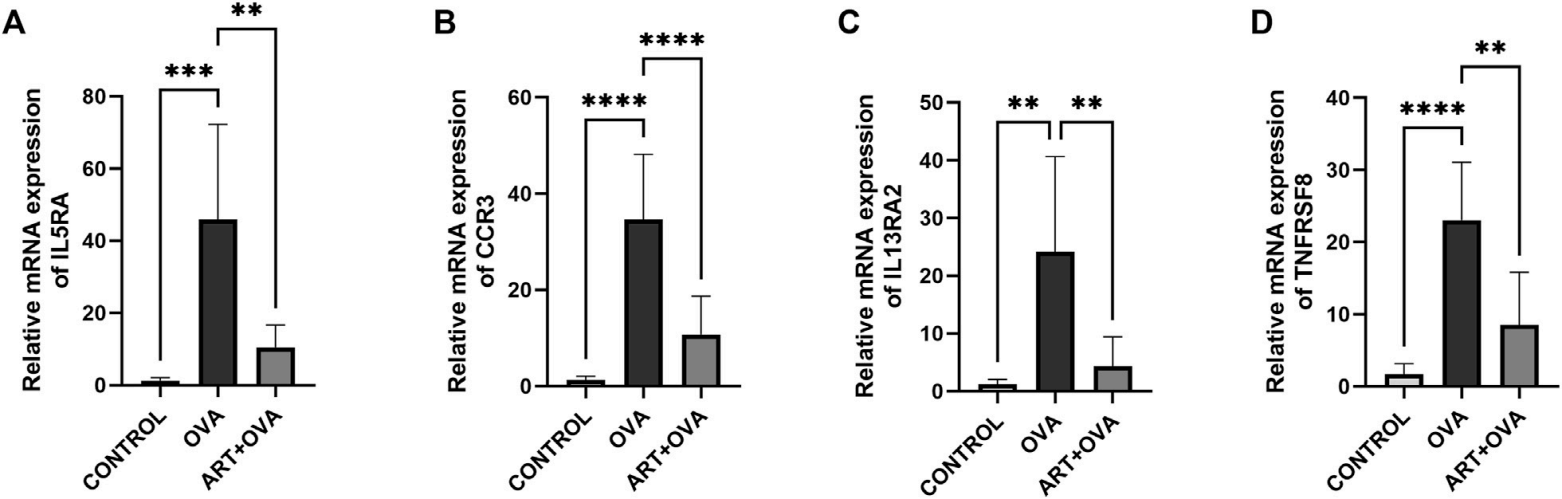
Effect of ART on OVA-induced asthma on mRNA expression levels. **(A–D)** Relative mRNA expression of IL5RA, CCR3, IL13RA2, TNFRSF8 in three groups: control, asthma (OVA), and ART + asthma (ART + OVA) group (*n* = 6). *p < 0.05, **p < 0.01, ***p < 0.001, ****p < 0.0001.

The credibility of results has been verified by selecting four genes (IL5RA, CCR3, IL13RA2, TNFRSF8) which were upregulated in Asthma groups, but downregulated in ART + Asthma groups, according to the log2 (fold change) value. And then analyzed their mRNA levels by qPCR. In the lung tissues of the animals with experimental allergic asthma induced by OVA, the IL5RA, CCR3, IL13RA2, and TNFRSF8 expression levels were considerably higher in comparison to the control group as illustrated in Figure 7. After treatment with ART, there was considerable downregulation in the expression. The mRNA levels were prominently upregulated in the model group, and ART treatment strongly reduced their upregulation. This demonstrated the credibility of this study’s findings.

### 3.6 ART alleviated FIZZ1 expression in asthma mice model

**FIGURE 8:**
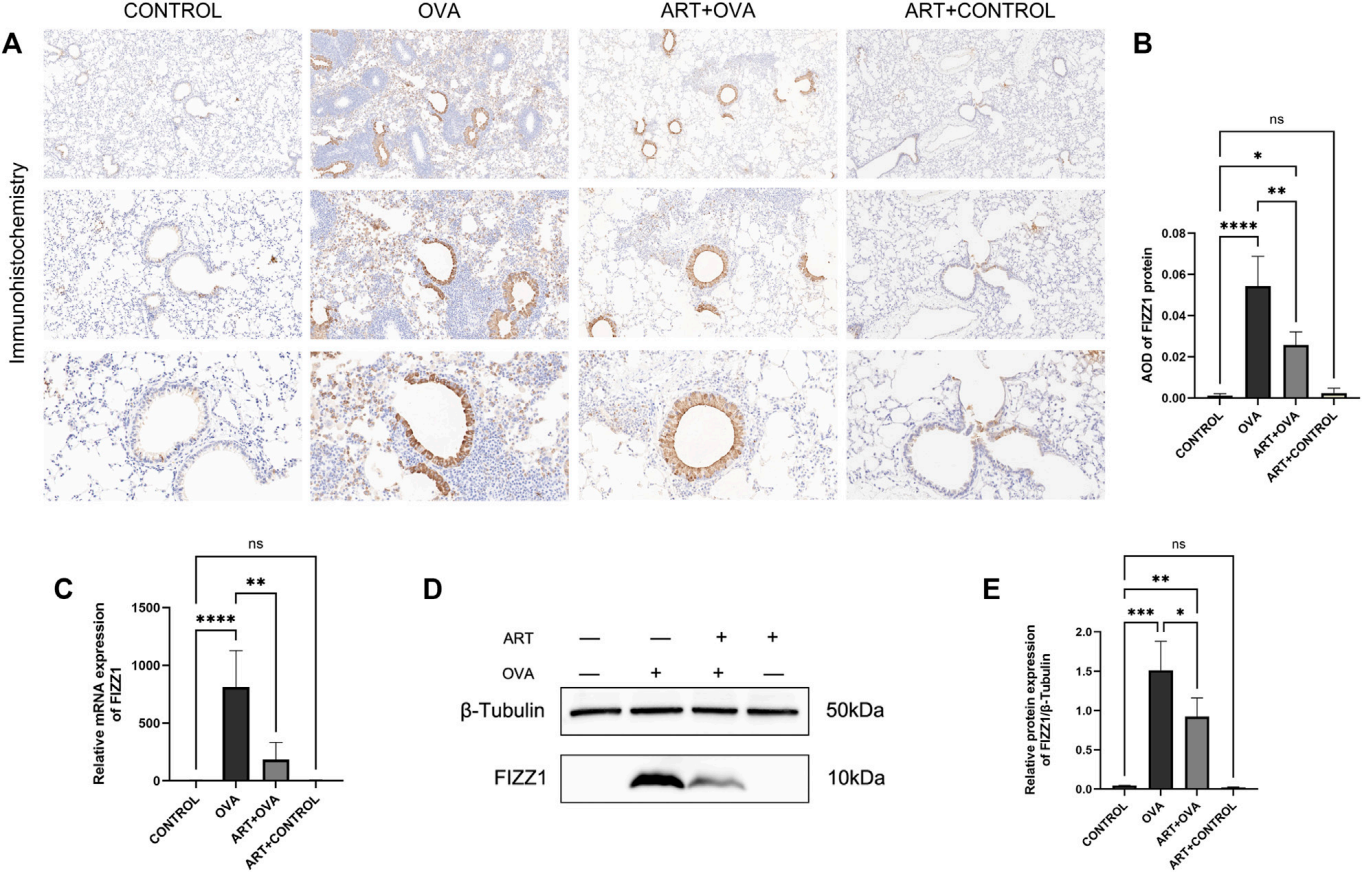
Effect of ART on FIZZ1 in OVA-induced asthma mice. **(A)** Representative immunohistochemistry photographs of FIZZ1 (*n* = 6) (Images were captured at ×100, ×200, and ×400 magnification). **(B)** The results are expressed as mean ± SD. AOD, average optical density. **(C)** Relative mRNA expression of FIZZ1 in four groups: control, asthma (OVA), ART + asthma (ART + OVA), and ART + CONTROL group (*n* = 6). **(D, E)** Western blot analysis of FIZZ1 (*β*-Tubulin was used as protein loading control) in the lungs from each group (*n* = 3). *p < 0.05, **p < 0.01, ***p < 0.001, ****p < 0.0001.

RETNLA, which also be called FIZZ1 (found in inflammatory zone 1), was found to be highly expressed **(** Figure 5A **)** in asthma-vs-control groups. In addition to being secreted by epithelial cells and eosinophils (Lin and Johns, 2020), alternatively activated macrophages (M2 macrophages) can also secret FIZZ1 (Gordon and Martinez, 2010), which contributed to airway remodeling (Dong et al., 2008; Wang et al., 2014; Zhao et al., 2015). Meanwhile, in a mouse model of asthma triggered by OVA, our research team discovered that ART can suppress M2 macrophage polarization in an OVA-induced asthma mice model (not yet published). Therefore, the effect of ART on FIZZ1 and subsequent airway remodeling clearly attracted us to do the follow-up study. To explore the potential effects of ART on airway remodeling in OVA-induced asthma, immunohistochemistry has been performed for examining FIZZ1 expression. FIZZ1 expression was mainly located in bronchial epithelium, according to immunohistochemistry, but it was also seen in alveolar macrophage, particularly in the lung tissues of the model group (Figure 8A). Additionally, the expression level of the OVA group was noticeably higher than that of the control group, but significantly lower following the ART intervention (Figures 8A, B). RT-qPCR, as well as Western blotting analyses, revealed that the expression level of FIZZ1 in the asthma lung tissues was increased in comparison to the control group but decreased after ART treatment (Figures 8C–E).

### 3.7 ART suppressed the MAPK signaling pathway

**FIGURE 9:**
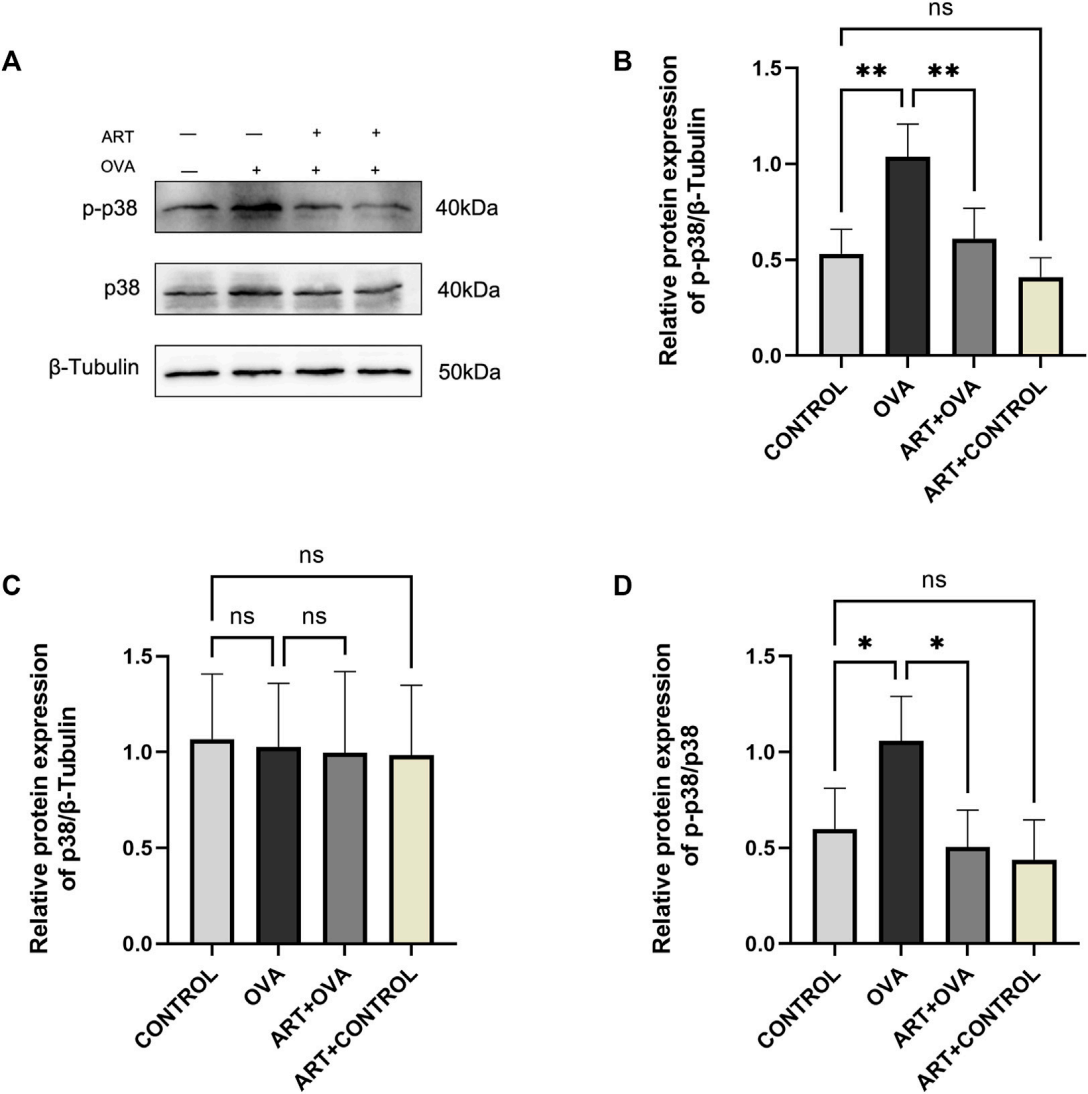
ART attenuated OVA-induced asthma by inhibiting p38 phosphorylation. **(A)** The expression of total p38 and phospho-p38 proteins in each group lungs (*n* = 4). **(B–D)** Quantitative analysis of protein levels in each group (*n* = 4). *p < 0.05, **p < 0.01.

The genes came into play by several signaling pathways, including cytokine-cytokine receptor interaction, PI3K-Akt signaling pathway, MAPK signaling pathway. The MAPK signaling pathway may play a major role based on transcriptome analysis. One of these pathways is the P38 MAPK signaling pathway, which is active in the form of phosphorylated p38 (p-p38) MAPK. Thickening of the sub-epithelial basement membrane and other structural alterations seemed to underlie airway remodeling, suggesting a role for p38 MAPK in asthma (Pelaia et al., 2020). Meanwhile, p38 MAPK-induced bronchial inflammation and remodeling significantly contributed to the development, persistence, and amplification of airflow limitation (Pelaia et al., 2021). Besides, our previous researches confirmed that ART can exert an effect in asthma by PI3K/AKT pathway. So, the protein levels of p-p38 MAPK and p38 MAPK have been detected in this study. As documented by the Western blot (Figure 9), the protein expression levels of p-p38 MAPK were considerably higher in the model group. However, a significant decrease in the expression of p-p38 MAPK has been observed following ART treatment. The levels of p38 MAPK had no changes. The data suggested that ART ameliorated asthma by suppressing the activation of the p38 MAPK pathway.

## 4 Discussion

Asthma is a very heterogeneous disease with different endotypes. It can be divided into type 2 (T2) high (Th2-high endotype) and T2 low inflammation (Salter et al., 2021). A precise but more expensive alternative to conventional corticosteroid therapies is monoclonal antibody biologic therapy (Salter et al., 2021). The lengthy course of treatment might not be financially feasible for some patients. Patients must frequently visit the hospital where the drug is available. In addition, the aforementioned medications cannot completely prevent airway remodeling. At the same time, airway remodeling occurs earlier and more severely in severe asthma than in mild-to-moderate asthma (James et al., 2009). It is urgent to seek for new and economic treatments to prevent the emergence or alleviate the progression of airway remodeling. The operation method of Artesunate included oral, intravenous, rectal, and intramuscular injection. There was no significant difference in drug utilization rate among the different methods (Karbwang et al., 1994; van Agtmael et al., 1999; Kitabi et al., 2021). Oral artesunate might be the optimum usage on the basis of its likely higher convenience and economic benefit.

A previous study showed that ART inhibited IgE-induced Syk and PLCγ1 phosphorylation, production of IP(3), and rise in cytosolic Ca^2+^ level in mast cells (Cheng et al., 2013). Furthermore, nuclear levels of nuclear factor erythroid-2-related factor 2 (Nrf2), oxidative damage markers 8-isoprostane, 8-hydroxy-2-deoxyguanosine, and 3-nitrotyrosine were considerably elevated following ART treatment (Ho et al., 2012). Meanwhile, ART reduced the area of *a*-Smooth muscle actin-positive cells and decreased cyclin D1 protein abundance, targeting airway smooth muscle cells (ASMCs) hyperplasia *via* the PI3K/Akt/p70(S6K) pathway (Tan et al., 2014). ART blocked epidermal growth factor-induced phosphorylation of Akt and its downstream substrates tuberin, p70S6 kinase, and 4E-binding protein 1, as well as transactivation of NF-κB in normal human bronchial epithelial cells (Cheng et al., 2011). ART also reduced traction force and induced an increase in [Ca^2+^] in the cultured arterial smooth muscle cells, which was mediated by TAS2R signaling in part (Wang R. Y. et al., 2019). In summary, ART can ameliorate experimental allergic airway inflammation, airway resistance, airway smooth muscle cell proliferation, oxidative lung damage, and mast cell degranulation *via* multiple pathways. There have been experiments conducted *in vivo* and *in vitro* in these studies. ART has been studied not only because of its economic value but also its potential immunomodulatory effect, playing a role in many diseases, particularly those related to allergies (Lin et al., 2022). However, the overall mechanism of ART on asthma is unelucidated.

In recent years, transcriptomics has emerged as a powerful tool for studying the action mechanisms of a drug (Li et al., 2022). The OVA-induced mouse model is a common experimental asthma model. Numerous literatures have shown that OVA-induced asthmatic model mice are more likely to develop allergic asthma, which was mainly associated with eosinophilic infiltration (Bruselle et al., 1997; Kanehiro et al., 2001; Wagner et al., 2007; Luo et al., 2015; Wang Y. et al., 2019; Wang R. Y. et al., 2021; Li et al., 2022). Allergic asthma was triggered by exogenetic allergens, presenting type two inflammation (Salter et al., 2021), and led to aggregation of eosinophils in BALF, paravascular and paratracheal region. The transcriptomic analysis has been applied to shed light on Artesunate’s protective mechanisms, which provided a more holistic understanding. Except for the mice behavioral change that was observed subjectively, the asthma model has been successful based on a series of objective indicators such as lung histopathology. With Artesunate intervention, airway inflammation, airway mucus secretion, and airway remodeling were found to be ameliorated, compared with the model group. It is suggested that artesunate exerts a protective effect on the eosinophilic predominantly asthma phenotype. Meanwhile, transcriptomic analysis was conducted to explore the overall regulatory effects of ART. In comparison to the OVA group, 2,323 DEGs have been identified among ART administrated asthma group of which 1,262 were upregulated and 1,061 were downregulated. It showed that ART’s effects on asthma are exerted through numerous genes rather than just one. Meanwhile, the genes involved, innate immune response, and inflammatory response by several signaling pathways, including cytokine-cytokine receptor interaction, PI3K-Akt signaling pathway, MAPK signaling pathway, and so on. Further, four genes including IL5RA, CCR3, IL13RA2, and TNFRSF8 have been selected to verify the accuracy and reliability of transcriptomic analysis by quantitative RT-PCR. Afterward, the PPI network was analyzed again using MCODE by Cytoscape. According to the relationship between edges and nodes in the huge network, the key subnetworks and genes can be found for downstream analysis, mainly including immune system, inflammation, or the remodeling process. The impact of artesunate on mast cells, eosinophils, and ASMCs has been investigated in the past. However, how artesunate affects alveolar epithelial cells biologically, for instance alveolar epithelial type II cells, which were shown to potentially contribute to the fibroproliferative response (Ruaro et al., 2021; Confalonieri et al., 2022). These are something that also piques our curiosity.

As seen on Figure 4A, asthmatic mice had higher levels of FIZZ1 expression than the control group. FIZZ1 was first discovered in experimental OVA-induced allergic pulmonary inflammation mice (Holcomb et al., 2000). Early research discovered that lung fibroblasts overexpressing recombinant FIZZ1 increased type I collagen and a-smooth muscle actin (Dong et al., 2008). And type I collagen and fibronectin-1, were able to express excessively *in vitro* when induced by the FIZZ1 recombination protein (Zhao et al., 2015), but their expression levels decreased when FIZZ1 was silenced. In addition to damaging the epithelial barrier, FIZZ1 could also alter the contraction property of tracheal smooth muscle by activating the mitogen-activated protein kinase pathway (Chen et al., 2010). FIZZ1 contributed to airway remodeling. After the intervention of ART, the FIZZ1 level was observed to be lowered compared with the model group. Immunohistochemistry showed that the protein was mainly located in epithelial cells, and the density in the airways epithelial cells layer was obvious. Whether this protein is involved in epithelial-mesenchymal transition needs to be realized by further studies. As a result of the aforementioned, ART may interfere with airway remodeling by controlling and alleviating the over expression of FIZZ1. IHC, WB, and RT-qPCR testing all revealed that FIZZ1 decreased after ART treatment. However, in contrast to the asthma group, FIZZ1 expression did not change according to the RNA-seq results in ART group. We hypothesized that this discrepancy might have been caused by the sample size restriction. This small episode cannot deny the crucial role of RNA-seq technology in revealing the global level of gene expression.

**FIGURE 10:**
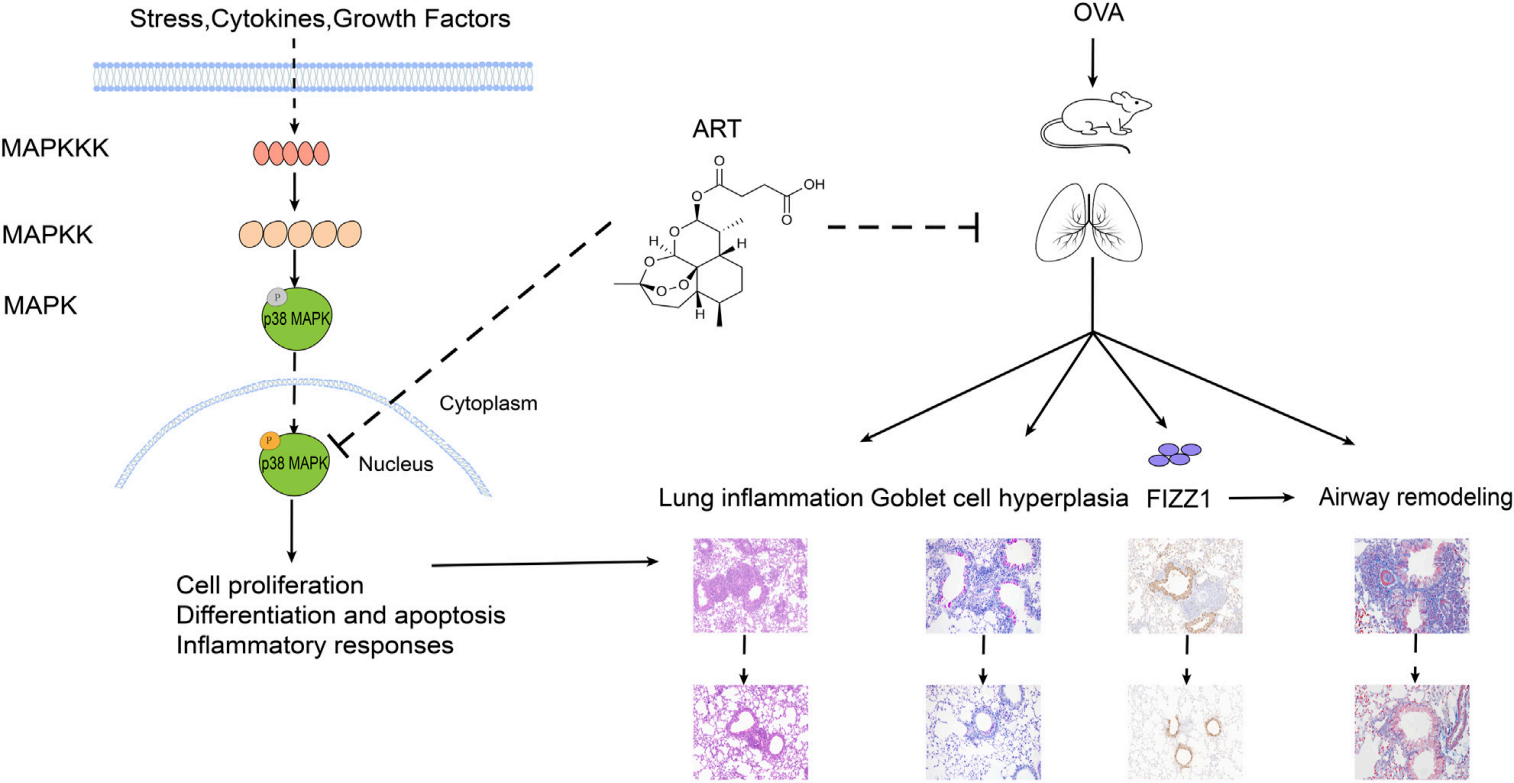
Potential mechanism of ART alleviating OVA-induced asthma by inhibiting p38 MAPK signaling pathway.

In the KEGG pathway enrichment, the MAPK signaling pathway was involved in ART + asthma-vs.-asthma groups. MAPK signaling pathway has four main branching routes: ERK, JNK, p38 MAPK, and ERK5 signaling pathway. The basic component of the MAPK pathway is a tertiary kinase pattern, including MAPK kinase kinase (MAPKKK), MAPK kinase (MAPKK), and MAPK. These three kinases can be activated sequentially. P38 MAPK signaling pathway is relatively simple and straight, with multilevel regulation, cascade amplification, and phosphorylation-based forms (Fang and Richardson, 2005). There was evidence that P38 MAPKs might respond to inflammation and stress as well as regulate proliferation, differentiation, and survival in certain types of cells (Cuadrado and Nebreda, 2010). Baines found that there was increased p38 signaling activity *via* induced sputum transcriptomics in severe asthma patients (Baines et al., 2020). Blocking p38 MAPK could effectively alleviate the exacerbation of allergic asthma induced by formaldehyde and high relative humidity (Duan et al., 2020). Meanwhile, p38 MAPK significantly contributed to bronchial inflammation and remodeling (Pelaia et al., 2021). It has been hypothesized that ART could improve asthma in part by regulating the p38 MAPK pathways. As result, ART indeed significantly inhibited P38 MAPK phosphorylation, which can induce downstream biological processes such as cell proliferation and differentiation, inflammatory response, then promotes the improvement of disease status (Figure 10).

This study provided a holistic genetic understanding of the role of ART in asthma, including gene functions, components, processes, and pathways. This study provided new theoretical evidence and potential pathways for the pharmacological mechanism of ART in improving asthma. But there were some limits. Firstly, the animal models are not fully representative of human disease. Besides, we only investigated the effect of ART on OVA-induced BALB/c asthma mice, and more animal models are needed, such as the HDM-induced asthma model or rat model.

## 5 Conclusion

This study confirmed the definite efficacy of ART in the treatment of asthma and revealed that ART can inhibit the expression of FIZZ1 protein and airway remodeling, which could act by inhibiting the p38 MAPK signaling pathway. Several genes and pathways have been implicated in the treatment of asthma by ART. The findings of this research add to the growing body of data supporting ART in treating asthma and provide the groundwork for its potential use in future clinical practice.

## Data Availability

The datasets presented in this study can be found in online repositories. The names of the repository/repositories and accession number(s) can be found in the article/supplementary material.
